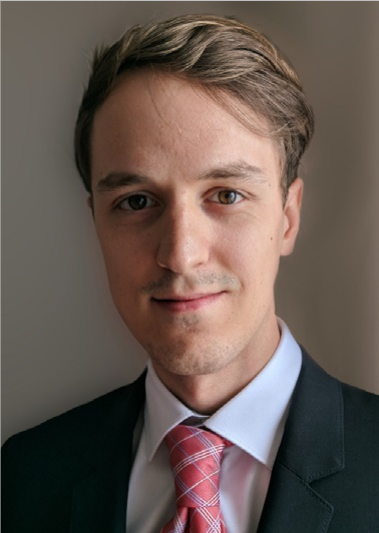# Beyond Frameworks: Structuring Reticular Materials across Nano‐, Meso‐, and Bulk Regimes

**DOI:** 10.1002/anie.201914461

**Published:** 2020-10-02

**Authors:** Frederik Haase, Patrick Hirschle, Ralph Freund, Shuhei Furukawa, Zhe Ji, Stefan Wuttke

**Affiliations:** ^1^ Institute for Integrated Cell-Material Sciences (WPI-iCeMS) Kyoto University, Yoshida, Sakyo-ku Kyoto 606-8501 Japan; ^2^ Department of Chemistry and Center for NanoScience (CeNS) Ludwig-Maximilians-Universität München Butenandtstrasse 11 81377 Munich Germany; ^3^ Department of Synthetic Chemistry and Biological Chemistry Graduate School of Engineering Kyoto University, Katsura, Nishikyo-ku Kyoto 615-8510 Japan; ^4^ Department of Chemistry Stanford University Stanford California 94305-5012 USA; ^5^ BCMaterials Basque Center for Materials UPV/EHU Science Park 48940 Leioa Spain; ^6^ Ikerbasque Basque Foundation for Science 48013 Bilbao Spain

**Keywords:** covalent organic frameworks, external control, metal-organic frameworks, nanoparticles, self-assembly

## Abstract

Reticular materials are of high interest for diverse applications, ranging from catalysis and separation to gas storage and drug delivery. These open, extended frameworks can be tailored to the intended application through crystal‐structure design. Implementing these materials in application settings, however, requires structuring beyond their lattices, to interface the functionality at the molecular level effectively with the macroscopic world. To overcome this barrier, efforts in expressing structural control across molecular, nano‐, meso‐, and bulk regimes is the essential next step. In this Review, we give an overview of recent advances in using self‐assembly as well as externally controlled tools to manufacture reticular materials over all the length scales. We predict that major research advances in deploying these two approaches will facilitate the use of reticular materials in addressing major needs of society.

## Graphical abstract

Structuring reticular materials beyond periodic lattices places an emphasis on the surface functionality, morphological manifestation, and the arrangement of crystal constituents at the final stage. By harnessing the power of self‐assembly and external tools, framework structures have been deliberately controlled across all length scales that excel in diverse application scenarios.

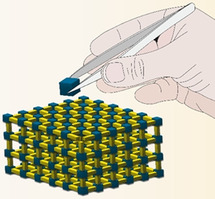

## 1 Introduction

Thousands of years of history highlight the great human achievements in shaping materials with manual, mechanical, and robotic tools. This control of material structure and morphology is widely practiced on length scales ranging from millimetres to kilometres, by using external tools with a size comparable with that of the materials themselves, thus making structural manipulation feasible. A significant effort has been centred on creating materials of smaller sizes to unlock the potential of new functions, such as large surface areas and fast charge transport. This objective has been greatly hindered by the difficulty in minimizing the tool–material interface, which is not completely addressed even with the advent of lithography techniques. Chemists, on the other hand, have embarked on accessing the “room at the bottom”,^[1]^ by piecing together molecular building units into chemical architectures under the principle of self‐assembly.^[2]^ The organization of molecules,^[3]^ polymers,^[4]^ and colloids^[5]^ as a consequence of specific, local interactions instead of external direction, has led to a plethora of structures being designed and synthesized particularly on the nanoscale.^[6]^ This synthetic effort, when dedicated to a larger size regime, especially with higher structural complexity and consequently advanced functions, has created a landscape of materials that is often referred to as mesomaterials.^[7]^ The power of self‐assembly into ordered structures at the bulk level, however, typically fails to fully manifest itself. A well‐planned strategy to transform the definitiveness of molecular interactions and bonding across multiple size regimes all the way into defined macroscopic objects is, therefore, required.

Within the frame of building‐up materials, reticular chemistry—linking molecular building blocks through strong chemical bonds into porous, crystalline framework structures—provides a high level of chemical control.^[8]^ Through reticular synthesis, organic molecules of well‐defined shape, geometry, and functionality can be assembled with each other into covalent organic frameworks (COFs),^[9]^ or with metal‐containing building units to form metal‐organic frameworks (MOFs).[^8a^, ^10^] The molecular nodes and struts of framework structures are designed in such a way that they remain rigid and directional during their reticulation, thus allowing for structure prediction and design. The connection of these sizable building units opens up the rigid framework backbone to create permanent pores.^[11]^ The accessibility of the interior of these materials to guests, in addition to their chemical designability, has led to tremendous research interest in reticular materials, especially for their use in gas adsorption, catalysis, sensing, and drug delivery.[^10b^, ^12^]

To meet the requirements of real‐world applications, the structure and morphology of reticular materials on the macroscopic length scale becomes an essential element for achieving optimized performance.^[13]^ The necessity of such design beyond the crystal structure is exemplified by packing particles into a monolith to maximize the volumetric efficiency.^[14]^ Additionally, creating interconnected channels within a monolith significantly reduces the pressure drop (the detrimental effect of resistance to flow), thereby facilitating fast kinetics in gas storage or separation.^[15]^ Aligning crystals on a 2‐dimensional (2D) substrate with defined orientation^[16]^ and thickness^[17]^ can lead to the desired anisotropic properties (e.g. conductivity, permeability, optical performance).

Controlling these macroscopic structural features is not straightforward, given that the synthetic repertoire available currently to reticular chemists is largely confined to the molecular regime. In fact, a common result in the synthesis of frameworks is polycrystalline power, which fails to exhibit well‐defined volumetric properties and anisotropic alignment. Efforts have been made to grow large single crystals to fill this design gap,^[18]^ but significant challenges are still present in the engineering of crystal growth and alignment. The unfavourable mechanical properties of framework single crystals further prohibit their direct use. Consequently, tools beyond crystal engineering are required to advance the manufacturing of reticular materials to a higher level of complexity and functionality. This budding field of research highlights the importance of physical designability in crafting reticular materials, which can be complementary to the chemical control harnessed by reticular synthesis. Here we adopt the concept of chemical control to describe the common practice of solvothermal synthesis, which is typically governed by thermodynamics. To highlight this aspect, we use the term “self‐assembly”, but do not infer that the connection between framework building units are weak, non‐covalent interactions. Instead, framework structures are distinguished from supramolecules by their strong chemical bonds. On the other hand, physical designability refers to the implementation of externally controlled tools, such as microfluids and 3D printing. It is noted that during the process of using external tools, chemical reactions can still take place, but not following the same energy pathway as their bulk‐state counterparts. Such external guidance imposes energetic influence on the growth of crystals or directs their spatial arrangement.

**Figure 1:**
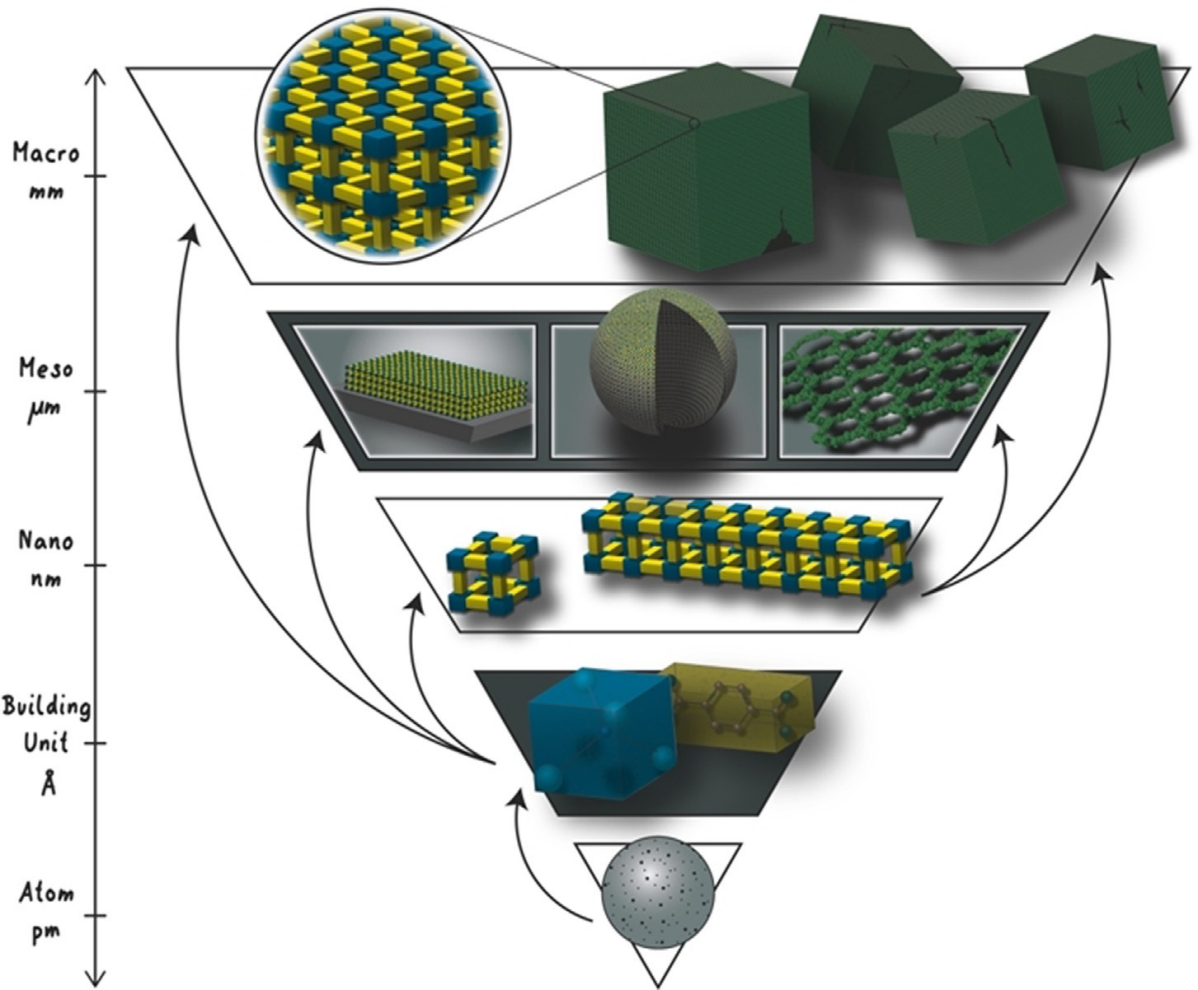
Building up reticular materials on all length scales. Atoms are used to construct secondary building units and atoms, which can be used to directly synthesise nano‐, meso‐, and bulk scale reticular materials. Additionally, meso‐ and bulk objects can be constructed by the assembly of nano‐objects.

In this Review, we give an overview of the experimental achievements in the structural and functional control of MOFs and COFs by highlighting three distinctive length scales—nano, meso, and bulk (Figure 1, Table 1, Figure 5). We hope to bring to the attention of the reticular community the importance of using external control to shape reticular materials, and its cooperative deployment with self‐assembly for structuring reticular materials in each size regime. For this purpose, the specific physiochemical properties arising from each length scale and how they relate to the performance in different application fields are discussed in detail. Finally, we propose strategies and tools that can be used for constructing reticular materials into larger size regimes with precise control over the structure, function, and morphology for direct use in real‐world applications. The insights gained across the different length scales can help address current limitations in framework synthesis and promises the future of reticular materials will have a broad impact on science, technology, and society.

**Table 1:** Important length scales and their associated chemical and physical parameters. At sizes over 1000 nm, these objects display the same properties as their macroscopic counterparts and are, therefore, considered bulk objects.

	Molecules	Nanostructures	Mesostructures	Bulk
Scale	1–10 Å	1–100 nm	100–1000 nm	>1000 nm^1^
				
Building blocks	atom	molecules	molecules/nanocrystals	molecules/nanocrystals/mesostructures
				
Features	functionality	size	crystal extension	shape, mechanical
	connectivity	surface	interface	density, heat/mass transfer
				
Synthesis approaches	self‐assembly	self‐assembly	self‐assembly or external control	external control

## 2 Chemical Designability of Reticular Materials

The crystal structure of reticular materials are programed and built at the molecular scale, where highly directional and rigid building blocks can be pre‐designed and synthesized by organic synthesis or metal complex chemistry.[^8a^, ^9b^] These building blocks are then joined to each other through chemical bonds in a reversible manner to create crystalline solids. The detailed knowledge of organic synthesis, which has accumulated over the last 200 years, essentially allows the synthesis of any stable molecular building block that can be imagined, only limited by the necessary time and resources. The rigid and directional nature of the building blocks makes the structural variation highly predictable compared to traditional solid‐state chemistry, one of the reasons for their wide promise for application. Organic linkers are synthesized with multiple attachment points to lead to extended solids, with typically two to four attachment points, but many examples exist with more.^[19]^ These attachment points consist of functional groups that coordinate with metal centres in MOFs,^[20]^ or create a covalent connection through dynamic covalent chemistry in COFs.^[21]^ The formation of metal‐containing units can be realized in an endless range of possible connectivities, sizes, and geometries, ranging from isolated clusters^[22]^ to infinitely extended one‐dimensional (1D) rods^[20]^ and 2D sheets.^[23]^ The geometry of the combined organic/inorganic building blocks defines the structure type of the final framework, thereby allowing the construction of a rich chemical space of materials by design. In COFs the linking groups are more limited in their geometries, with most employed COF linkages being linear.^[9b]^

The power of chemical designability in reticular materials not only comes from the rich chemistry and the many possibilities of combining building blocks, but also from the ability to selectively vary a single structural parameter or functional group without completely changing other parameters of a structure, thus allowing the desired properties of the framework to be tuned for the corresponding applications.^[24]^ This is exemplified by the concept of isoreticular substitution, which describes the synthesis of a framework with the same overall structure, nets, and often metal nodes but with different linkers.^[25]^ The ability to change the linker without altering the net leads to detailed control over the properties of the materials, and allows rational design of structures.[^24a^, ^24b^, ^26^]

**Figure 2:**
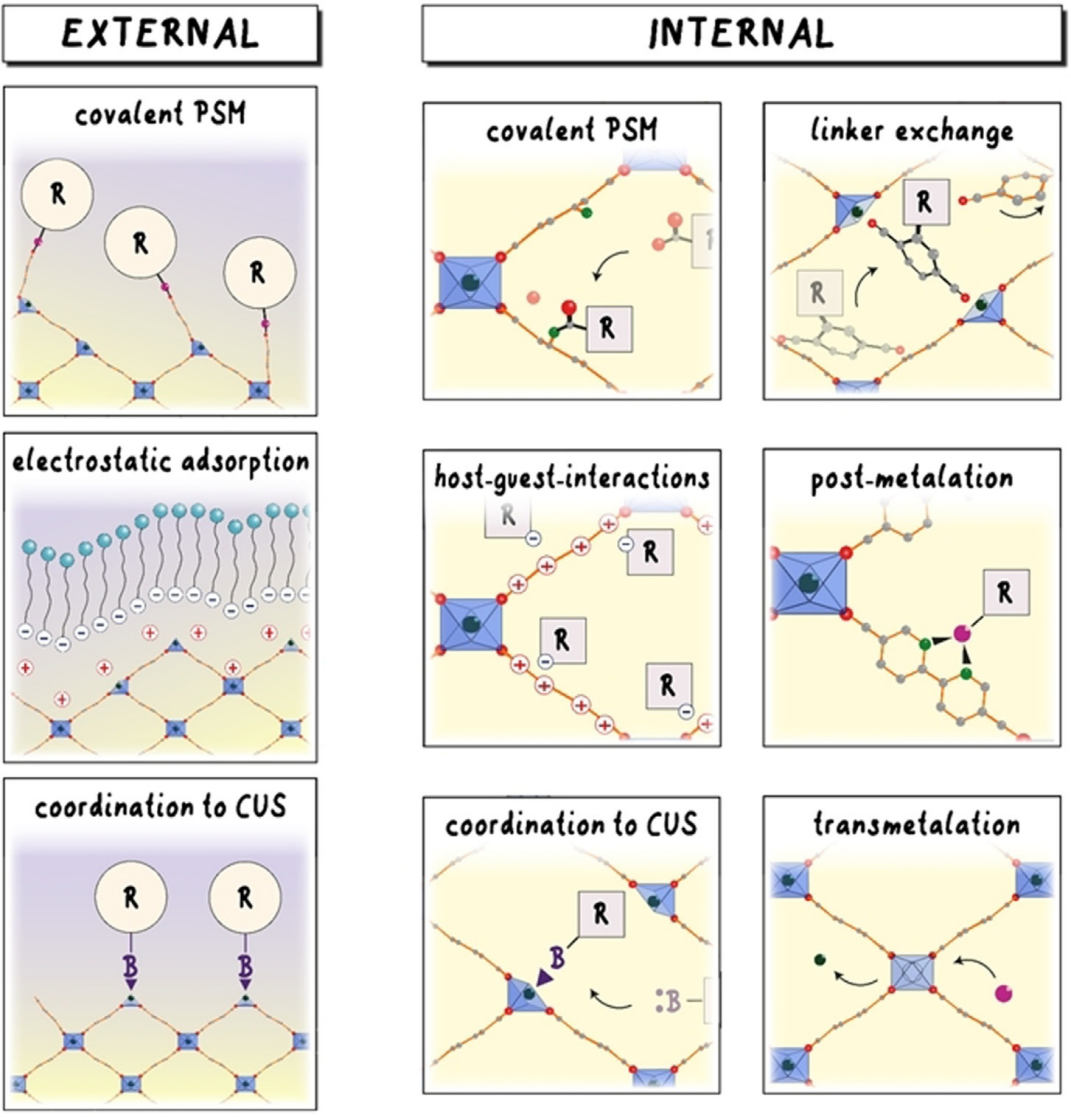
Different post‐synthetic modification strategies (PSMs) used for the introduction of functionality. There are two general concepts: selective functionalization of the external surface and the internal modification of the periodic lattice. B stands for a coordinating linker. R can be any residue.

The rigidity and directionality of the molecular building blocks create free permanent voids, thus making these materials porous.^[9b]^ It is possible to functionalize reticular materials on the internal surface, that is, on the surface of interior pores, and also on the external surface, which is defined as the limit of the periodic structure terminated by facets of a crystallite or the interface with the surrounding medium.^[27]^ These fundamentally different approaches, termed internal and external modification (Figure 2), respectively, allow the properties of reticular materials to be modulated to meet the needs of specific applications.

General approaches to the modification of MOFs and COFs introduce desired functionalities to organic linkers and metal nodes through the formation of new strong interactions with the framework backbone (Figure 2).^[28]^ These can be by introducing a covalent bond to the linker, ionic interactions between the framework backbone and guest species, a coordination bond formed by binding new ligands to coordinatively unsaturated sites (CUS) of metal nodes, or additional metals to linkers.[^27a^, ^27c^] Methods specific to internal and external modification will be covered in detail below.

The chemical designability has an immediate effect on the properties of the materials. Typical design parameters are the size and geometry of the framework pores, which are of importance to porosity and the adsorption of guest molecules. In MOFs, the metals carry much of the functionality of the framework, and can lead to the material having catalytic,^[29]^ optical,^[30]^ and magnetic^[31]^ properties, and also serve as selective adsorption sites.^[32]^ In COFs and MOFs, the linker is also used to influence the properties. The linkers of a framework can be used to modulate the optical properties,^[33]^ adsorption properties,^[34]^ work as catalysts,^[35]^ or serve as anchoring points for additional functional groups.[^26c^, ^28d^] Often, tuning the linker ensemble can be used to fine‐tune the guest adsorption^[36]^ or the catalytic performance of a pore‐mounted catalyst.^[37]^ Linker interactions are of special importance in controlling the conductivity^[38]^ and the opto‐electronic properties.[^24b^, ^39^] The chemical designability also affects important parameters for application, such as chemical stability.[^28e^, ^40^]

## 3 Internal Modification

The typical approach to modification of a framework is the chemical alteration of reactive moieties present within the crystal.[^27a^, ^28c^] During internal modification, the reactive moieties are accessible to incoming reagents that diffuse across interconnected pores, thus enabling the chemical reaction to take place. These functional groups are often deliberately included in the framework design to provide anchoring sites for post‐synthetic modification (Figure 2). The robust framework backbone ideally allows for structural modifications without compromising the integrity of the crystalline architecture. Other than addressing reactive moieties of the building units, functionalization strategies such as linker exchange^[41]^ and transmetalation^[42]^ are employed to substitute the whole building units with new types of building blocks or those with functionalities of interest. A special type of internal modification is post‐metalation, where metal species are introduced post‐synthetically to defined binding sites in the framework. In COFs, the post‐metalation of linkers is very important for the introduction of functionality;[^28e^, ^43^] this approach has also been used with MOFs.^[44]^

Most strategies to modify the internal surfaces of reticular materials aim to uniformly modify the properties of the parent framework materials. However, it is also possible to create structural changes beyond the periodic structure, such as creating a functionality gradient across a crystal. The heterogeneous modification can be facilitated by using light‐induced reactions that allow localized post‐synthetic modification. The thiol‐ene click reaction has been used to cross‐link the organic linkers of a MOF.^[45]^ This approach allows functionally graded materials to be produced or composites with abrupt changes in properties from the initially uniform materials, but it can also be used to selectively dissolve the non‐cross‐linked MOF to obtain structured gels, based on the previous patterning. The inherent diffusion limitation of post‐synthetic reactions, as they start at the faces of a crystallite that are in contact with the solvent and then react further into the inside of the crystallite, can be used to structure heterogeneous reticular crystals. This effect has been demonstrated in post‐synthetic topotactic linker exchange and has been visualized by fluorescence and Raman microscopy,^[46]^ and can be used to generate well‐defined core–shell structures.^[47]^

Internal post‐synthetic modification is effective in tuning bulk characteristics such as porosity^[48]^ as well as magnetic,^[49]^ optical,^[50]^ and electronical properties.[^49^, ^51^] It can also be used to modulate the flexibility^[52]^ of a structure, the mechanical properties,^[53]^ and change its polarity.^[54]^ Post‐synthetic modification is mostly used to tether functional groups to the inner pore surface,^[55]^ such as biologically active groups,^[55g]^ ionic functional groups,^[56]^ catalysts,^[55c]^ and gas‐releasing agents.^[57]^ This allows the introduction of fundamentally new properties to the reticular material or the stabilization of tethered guests that would otherwise deactivate in solution.^[58]^ In addition to the designed and desired changes in properties, a reduction in pore size and porosity is often observed.^[27a]^ In COFs, post‐synthetic modification of the linkage that connects the building blocks is used to alter the chemical and physical properties of the framework. The most drastic effect was seen in the stability of the frameworks, where this strategy shows promise for generating ultra‐stable frameworks that are much more stable than most MOFs.[^40^, ^41^, ^59^]

## 4 External Modification

External modification encompasses the chemistry at the 2D interface between a reticular structure and its surrounding medium, mostly performed on nanoparticles and mesostructured materials because of their large ratio of outer surface area to volume.[^27b^, ^60^] Two strategies have been developed to specifically functionalize the outer surface, but not the internal surface of reticular materials: by targeting reactive sites present exclusively on the outer surface[^27b^, ^61^] or confining reactions through steric hindrance.[^31^, ^62^] The former strategy takes advantage of the accumulation of coordinatively unsaturated metal sites^[63]^ or dangling terminal linkers on the external surface.^[64]^ The success of the latter strategy relies on a molecular‐sieving effect, which precludes the penetration of reagents into the crystal interior.^[65]^ The surface confinement of the modification reaction has also been enabled by hydrophilic/hydrophobic phase separation,^[66]^ electrostatic adsorption,^[67]^ and covalent binding.^[68]^ External modification enables the modulation of the external surface polarity of nanoparticles by introducing hydrophobic coatings, which leads to improved chemical stability in water and colloidal stability in non‐polar solvents.^[69]^ Fluorescent molecules are also frequently introduced to label framework nanoparticles for bio‐imaging purposes.[^27b^, ^70^]

## 5 Physical Designability beyond Crystal Structure

An important limitation of self‐assembly is the difficulty in controlling the physical properties of the final material, for example, mechanical performances and morphology. Therefore, there has been significant interest in applying externally controlled tools that offer accurate spatial control to shape materials over multiple length scales.^[71]^

Many of these techniques using external control for structuring solids originate from the field of micro‐ and nanofabrication technology developed for microelectronics, which has branched and spawned across many new areas such as microfluidics,^[72]^ micro‐optics,^[73]^ and micro‐electromechanical systems for reticular materials.^[74]^ Although the number of applications and their diversity have multiplied over the years, the pool of materials that are compatible with routine micro‐ and nanofabrication processes has long been dominated by inorganic dielectrics and semiconductors. We envision that this limitation will be overcome by a range of new and highly versatile fabrication technologies that may vastly expand the choice of materials and have a continuing impact on the manufacture of reticular materials.

**Figure 3:**
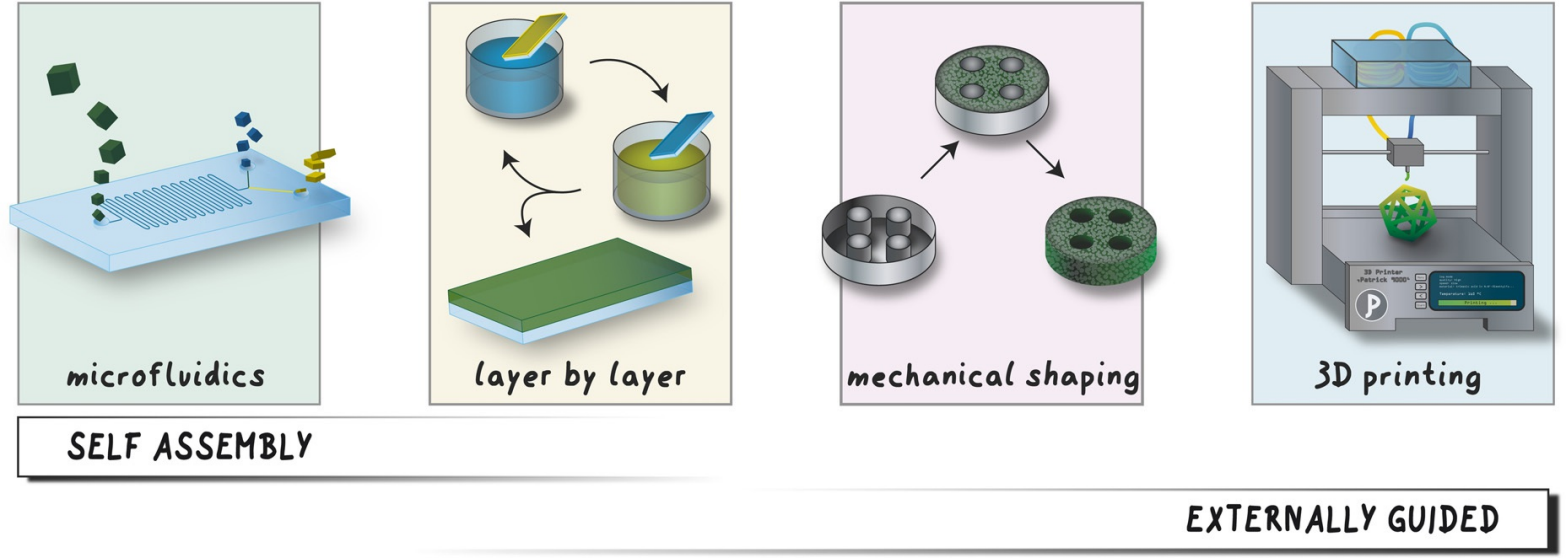
The interplay between self‐assembly and external control in synthetic approaches to structuring reticular materials.

The future development of physical control over reticular materials also relies on the advancement of additive manufacturing techniques, such as 3D printing,^[75]^ two‐photon polymerization,^[76]^ and focused ion‐beam deposition.^[77]^ These techniques are becoming mature and widely available. Many subtractive methods, such as ultra‐short pulsed laser ablation have been or are becoming available for the processing of MOFs and COFs, such as nanotemplating techniques, mechanical shaping methods, and 3D printing (Figure 3).^[78]^

**Figure 4:**
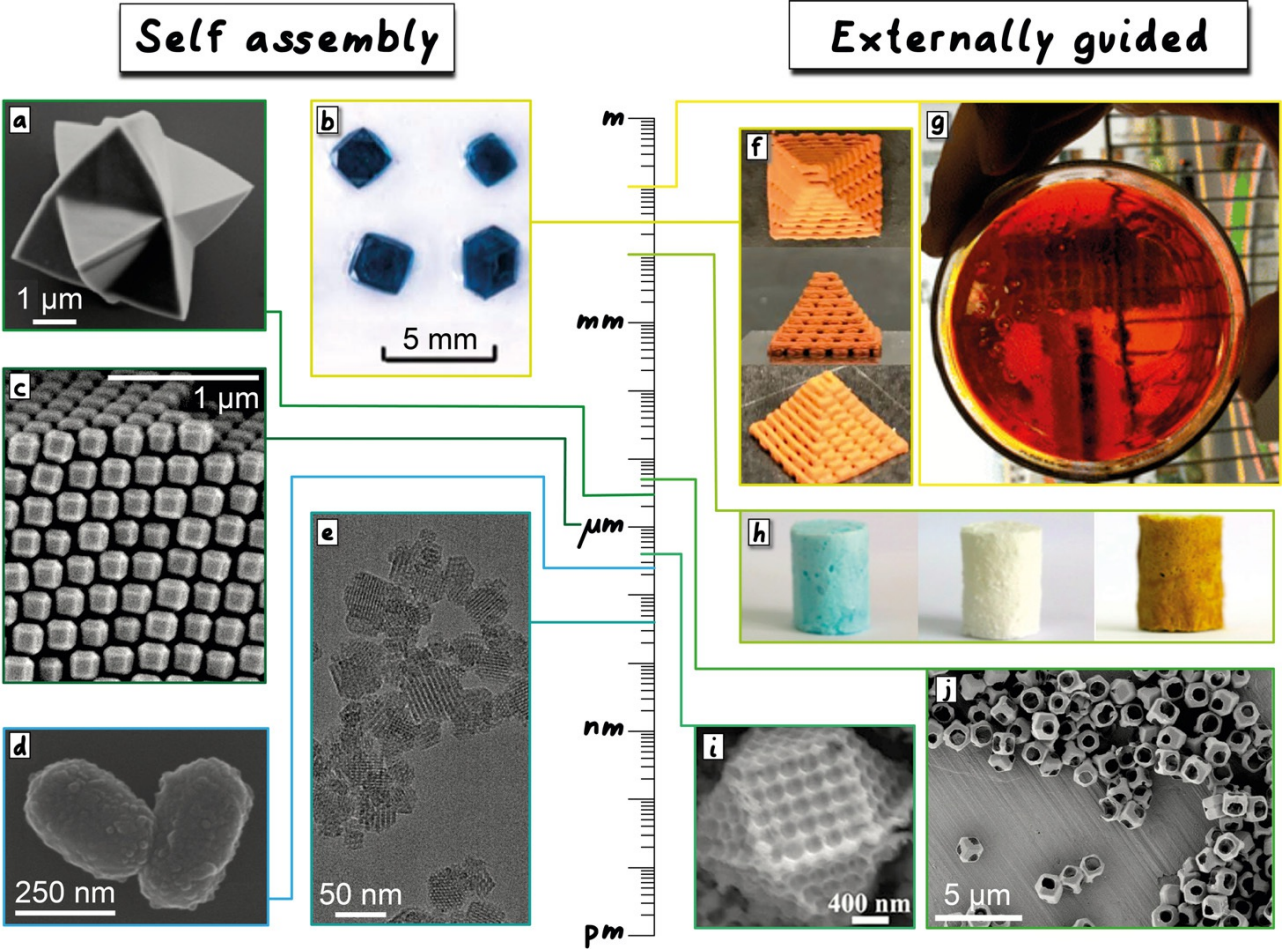
Examples of reticular objects synthesized over a wide range of length scales through either self‐assembly (left) or external guidance (right). a) CuBr(4,4′,4′′,4′′‐tetrakis(4‐pyridylethen‐2‐yl)tetraphenylmethane) microstars (solvothermal),^[98]^ b) millimetre‐sized Cu_3_(BTC)_2_ crystals (solvothermal),^[99]^ c) assembly of truncated rhombic dodecahedral ZIF‐8 nanoparticles (evaporation),^[100]^ d) MIL‐101(Cr) nanoparticles (microwave),^[28a]^ e) PCN‐222‐Co@TpPa‐1 MOF@COF nanoparticles (solvothermal),^[101]^ f) TPE‐COF‐precursor/F127‐ink pyramid (3D printing),^[81a]^ g) MOF glass corresponding to Ti_16_O_16_(BPA)_*x*_(OR)_32−*x*_(*x*≈4, OR=cresolate, hydroxide, ethoxide) in a petri dish (evaporation of plasticizer‐modulator solvent),^[102]^ h) HKUST‐1, ZIF‐8, and NH_2_‐UiO‐66 based foams (freeze‐drying),^[103]^ i) a single crystal of ZIF‐8 with ordered macropores (templating),^[79]^ and j) ZIF‐67 microcubes (etching).^[104]^

A central aspect in the physical designability is the external control, which is used to position and shape reticular materials. External control over MOFs and COFs can be exerted by many different means, ranging from casting into template shapes[^18a^, ^79^] (Figure 4 i), direct ink writing into 2D^[80]^ and 3D^[81]^ architectures (Figure 4 f), whereby a reticular material is selectively added at a desired position, to dip pen lithography, where particles can be grown in confined volumes.^[82]^ The growth of particles in microfluidic channels is similar, where either the extent of the channel limits the growth^[18b]^ or particles are grown in droplets flowing in the microfluidic channel.^[83]^ The growth of thin films exploit nucleation^[16]^ and epitaxial growth on sacrificial substrates.^[84]^ Additionally, the growth of MOF films on self‐assembled monolayer surfaces allows precise control over their thickness.^[85]^ These techniques balance the self‐assembly and the externally guided processes to achieve a structured material.

The challenges that arise with externally controlled tools are the minimum feature size, which limit the accuracy and level of detail, and the possibility for scale‐up, which determines how much material can be produced by these methods. Often these two features are inversely related to each other, where accuracy, complexity, and small feature size are in opposition to the amount of product that can be patterned. Self‐assembly can help in producing structuring at a length scale that cannot be achieved with an externally guided method. Methods based on self‐assembly can be scaled up more easily.

The methods used to alter the properties at different length scales differ significantly, with smaller structural features being dominated by methods controlled by self‐assembly and larger structural features by externally guided methods (Figure 3). When multiple size regimes are affected at the same time, they are often achieved by a combination of self‐assembly at small length scales and an externally guided method at larger length scales.^[86]^ Different size regimes can therefore be addressed independently.

## 6 Reticular Nano‐Objects

Decreasing the size of particles leads to a drastic increase in their exterior surface area relative to their volume, which is the defining characteristic of nanosized frameworks. As the key parameter of reticular materials in the nanoregime, the determination of particle size is particularly important and generally not trivial: different specific physical characterization techniques yield different results as they describe either the crystallite size, particle size, or hydrodynamic radius.^[87]^ In addition to reducing the diameter of a particle, another key criterion for realizing the promise of extensive surface areas requires the particles to be isolated and handled as well‐dispersed solutions.

Nanoparticles (Figure 4 d,e) of reticular materials naturally inherit the bulk properties such as ultra‐high porosity and internal surface areas, but display new properties that are dominant on the nanoscale, including short diffusion distances across the whole particle, abundant surface defects, facile chemical functionalization, cooperative structural dynamics, and higher chemical reactivity.[^6^, ^88^]

Decreasing the size of reticular materials to the nanoregime can increase the adsorption kinetics, whereas interparticle mesopores and non‐closing hysteresis loops in sorption isotherms can be caused by nanoparticle aggregation.^[89]^ When MOF nanoparticles are used as catalysts, the conversion rates can increase significantly compared to the bulk counterpart, because of enhanced diffusion into the particles making all the particle's inside available to function as a catalyst. Alternately, a larger outer surface area can lead to improved catalytic activity when active sites reside on the external surface.^[29]^

Small crystal sizes can also bring about a new dynamic behaviour: The adsorption of guest molecules can result in reversible changes in the crystal structure of a reticular material: open‐framework structures can be stabilized by guest molecules, while the removal of guest molecules can lead to closed non‐porous forms.^[90]^ The downscaling of the crystals to the nanoscale additionally gives access to transient, otherwise inaccessible, metastable structures, such as open frameworks, even after guest removal.^[91]^

**Figure 5:**
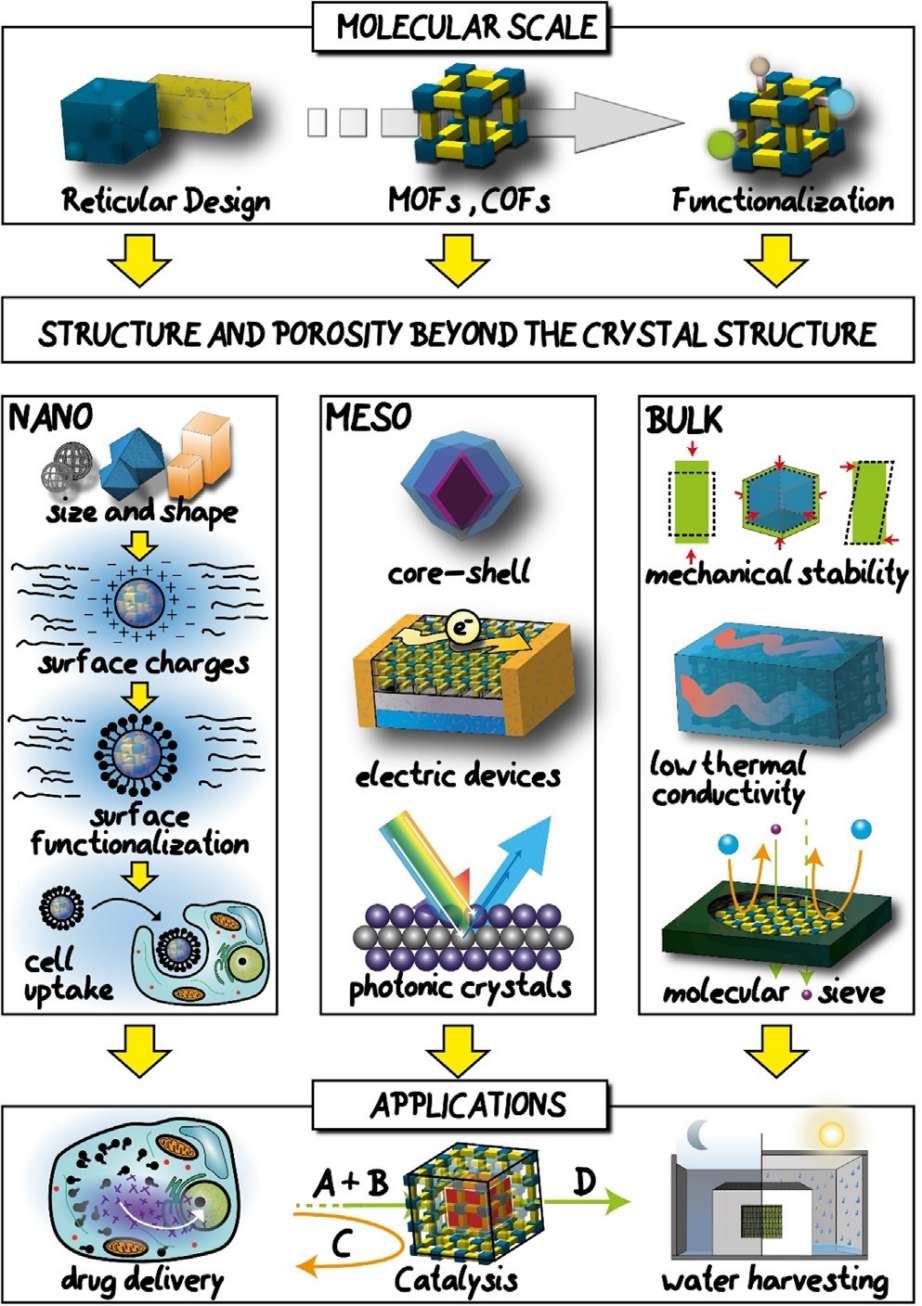
The concept of going beyond the crystal structure of reticular materials. Although many features of reticular materials can be designed in crystal engineering, their successful use in specialized applications require further optimization, namely nano‐ and mesostructuring as well as bulk processing.

The tailorable nature of the individual building blocks of reticular materials provides them with a high functionalization efficiency, which can be increased even further for nanospecies. Grafting different functional groups on the external surface of framework nanoparticles can introduce multivariate functionalities carried by a single particle to the surface.^[92]^ These functionalities—such as being fluorescent, magnetic, highly charged, and having molecule recognition sites—which can operate both individually and synergistically, thus allow one sophisticated nanoparticle to perform multiple tasks required for demanding applications such as drug delivery (Figure 5).

The synthesis of reticular materials in the nanoregime is mostly performed by self‐assembly (Figure 4). In these techniques, a precise optimization of the precursor concentration, feed rate, reaction temperature, and reaction time is necessary to control both the nucleation and growth of framework crystals to achieve narrow size distributions.^[93]^ Often modulators and surfactants are employed to gain control over the size of the particles.^[94]^ These modulators can be, for example, monofunctional derivatives of the linkers, surfactants, or polymers.^[95]^ Modulators enable the formation of nanoparticles by inhibiting growth, slowing down the reaction, and by inhibiting aggregation in solution, thus creating stable dispersions and allowing control over the size of the particles. Modulators further enable the stabilization of the nanoparticles in solution and thus inhibit aggregation, which would otherwise lead to undesired particle sizes and instability of colloidal solutions.^[96]^ Modulators can also be used to control the facets present on the nanoparticles, and tuning the modulator type and concentration can yield objects with increasing size and morphologies ranging from nanoparticles to 1D or 2D meso‐objects.^[97]^

Similar to molecular systems, the purification and recycling of colloidal dispersions of reticular materials is possible through techniques such as centrifugation, dialysis, and filtration, which effectively remove excess reactants, guests, and reaction products after catalytic reactions. These separation methods can also be applied to purify framework nanoparticles according to their sizes and functionalities.

## 7 Reticular Meso‐Objects

Reticular structures at a length scale of 100 nm to 1000 nm is no longer governed by principles of the nanoscale, and their properties are typically also not accessible in the bulk. Reticular materials on this meso length scale are defined by the size of their characteristic structural features (minimal fragment of the structure that defines the property of the material). For example, the characteristic feature of a membrane is its permeation pathway; therefore, a membrane on the mesoscale is defined by its thickness even though it may possess extensive lateral dimensions. At this length scale, the properties exhibited by the material are often affected by the interplay of multiple components of the structure, such as the alignment of constituent crystallites in their assemblies or the combined sieving effects between different layers in core–shell structures (Figure 5). Meso objects bridge the world of nano‐ and bulk materials and are able to combine the features common to both. On one hand, meso‐objects such as thin films display high external surface areas commonly found in nanomaterials.^[105]^ On the other hand, meso‐objects are optimized for performances that are characteristic of bulk materials, such as conductivity over a large length scale.^[106]^

Typical meso‐objects are structured such that the morphology and spatial arrangement of subunits can lead to the enhancement of properties or the emergence of new ones that cannot be derived from the crystal structure alone. Meso structures can be synthesized in the form of single or polycrystals through self‐assembly, externally directed approaches, or a combination of both (Figure 4 a).^[107]^ The synthesized mesostructures are classified here depending on their overall dimensionality: zero‐dimensional (0D, Figure 4 a i,j), 1D, 2D, and three‐dimensional (3D, Figure 4 c) systems.

0D meso‐objects often combine the structure features of nanoparticles with a higher level of complexity, such as compartmentalization. Two of the most common examples of this class are hollow particles and core–shell structures. Manufacturing these MOF and COF mesostructures has been realized by a large number of synthesis methods.^[108]^ In self‐assembly‐based approaches, the morphology of these reticular materials can be controlled through surface‐energy‐driven mechanisms (Figure 4 a),[^98^, ^108f^] modulating surfactants,^[109]^ or self‐templating.[^107e^, ^110^] Methods based on external control include etching[^104^, ^111^] and templating, which is based on the heterogeneous nucleation and assembly on the surface of the template and the replication of the template morphology.[^107a^, ^107b^, ^107c^, ^107d^, ^112^] Typically, these templates are then removed in a subsequent step to yield core–shell structures.^[113]^ When using reticular structures themselves as templates, it is possible to generate mesostructures with multiple layers, with each layer comprising a distinct framework.[^112c^, ^114^]

1D mesostructures can exhibit helical, fibrous,^[115]^ and needle‐shaped morphologies. Although there are examples of generating reticular 1D materials with specialized methods such as electrospinning^[116]^ or using external electric fields to produce 1D particle strings,^[117]^ these materials are typically synthesised by templated crystallization.^[118]^ This process can be carried out in the hollow channels of a hard template followed by template dissolution.^[119]^ Alternatively, this can be achieved by heterogeneous nucleation on 1D objects, facilitated by surface groups that induce crystallisation^[120]^ Other approaches use sacrificial templates to grow 1D MOF structures on surfaces.^[121]^ Similar to 0D mesostructures, the templated synthesis of 1D reticular materials allows the transfer of template morphology to the resulting meso‐object, which can either be used to increase the external surface area of the materials^[121]^ or to introduce specific functionality, such as chirality.^[120a]^

2D mesostructures include free‐standing membranes and surface‐supported films. A straightforward method of producing thin films and thin‐film heterostructures is the deposition of nanoparticle suspensions on a substrate by spin or dip coating.^[122]^ This method circumvents the necessity for nucleation and growth on the surface, but it is limited by the necessity to synthesise nanoparticles. Another approach is the direct growth of reticular materials on the surface of a substrate from a mother liquor/synthesis solution using the nucleation at the surface of the substrate for the initiation of growth.^[122]^ Variations of this direct growth have been used for the synthesis of MOF and COF thin films. A notable extension of this method is the vapour‐assisted growth of reticular thin films, where the reactive precursors are deposited on the substrate as a small droplet and the reaction mixture is heated in solvent vapour.[^105b^, ^105c^, ^123^]

The growth of MOFs and COFs can lead to oriented growth even in the absence of special functional groups at the surface.^[38b]^ However, surface‐supported reticular materials are often grown on surfaces that are functionalized to facilitate the growth of the crystalline materials and to improve the adhesion between the reticular material and the substrate.^[105a]^ For example, self‐assembled monolayers with functional groups such as carboxylic or amine moieties can undergo crystal growth on many different surfaces and can even direct the crystallographic orientation of MOF and COF films.[^85c^, ^124^] Films of 2D COFs are often grown on highly oriented pyrolytic graphene (HOPG) or graphene, as this substrate facilitates π‐π interactions between the aromatic backbone of the COF and the graphene, thereby leading to oriented growth.[^24a^, ^125^] Under well‐defined conditions, patterned single‐layer graphene on SiO_2_ can be used to achieve selective nucleation on graphene and thereby lead to patterned COF surfaces.^[126]^

The growth of reticular materials can be highly controlled by using a SAM in combination with a layer‐by‐layer growth or liquid‐phase epitaxy (LPE) to obtain highly oriented surface‐anchored MOFs (SURMOFs).^[78a]^ These thin films are obtained by the controlled sequence of adding the precursors, whereby a single layer of linker or metal precursor are deposited. The alternation of both steps with intermittent washing steps allows fine‐tuning of the height of the films, and allows the sequential deposition of MOF‐on‐MOF heteroepitaxial films.^[127]^ In a modified approach, 2D MOFs are synthesized by a Langmuir–Blodget approach and then transferred layer‐by‐layer to a substrate.[^105d^, ^106^] The deposition of MOFs by chemical vapour deposition has recently emerged as a method that is compatible with standard microelectronic structuring techniques, where a dense metal oxide, deposited in a first step, is transformed into a porous ZIF by reaction with methylimidazole vapour.^[105e]^

One interesting outcome of growing thin films on substrate surfaces is interfacial strain.^[128]^ In the case of MOF nanofilms, this can be used to access otherwise only metastable structures with lattice parameters deviating from the default values of their bulk counterpart, modulated by the magnitude of the strain. The structural distortion can lead to an increase in the symmetry of the crystal structure of the MOF film^[129]^ and stabilization of non‐interpenetrated structures.^[130]^ Synthesis on a 2D substrate allows for controlled growth of framework structures perpendicular to the substrate plane into the nanoscale. Such spatial confinement results in thin films that feature short diffusion distances. Additionally, compared to the bulk equivalent, MOF thin films present a high concentration of coordinatively unsaturated metal sites at the extensive interface with the adjacent medium.^[131]^ Reticular thin films further display increased structural rigidity, which can inhibit the expansion of the crystal structure upon guest uptake, a behaviour called “breathing”, thus leading to unique sorption properties.^[132]^

Free‐standing reticular 2D films can be manufactured by externally controlled exfoliation of pre‐synthesized crystals of stacked 2D layers into well‐dispersed solutions.[^105b^, ^133^] This process can be assisted by mechanical techniques,^[134]^ sonication,^[135]^ and chemical modifications. Facile exfoliation was enabled by introducing bulky functional groups to a COF backbone^[136]^ that decrease the interlayer interactions. Another example is to replace bridging linkers with non‐bridging ligands between layers, thereby leading to exfoliation of the MOF.^[137]^ In recent years, free‐standing 2D reticular films have also been synthesised through self‐assembly at the interface^[138]^ or in bulk solution.^[139]^

3D mesostructures, which are often called superstructures, exhibit a hierarchical arrangement of morphologically distinguishable components, which differentiates them from bulk materials.^[108b]^ They have been realized in the form of both single objects or extended assemblies of reticular components. 3D mesostructures have been synthesised by self‐assembling objects with lower dimensionality, such as nanoparticles,[^100^, ^140^] 1D helices,^[120a]^ and those capable of self‐templating.^[141]^ External means to produce 3D mesostructures include templating,[^79^, ^142^] and manually stacking thin‐film layers into 3D architectures.[^122^, ^138d^] The ordered arrangement of reticular constituents into superstructures can provide the resulting material with superordinate pores and other large cavities,^[143]^ thereby leading to better accessibility for guest molecules. The 3D mesostructuring of reticular materials can additionally increase the mechanical stability (Figure 5) and provide improved flexibility.^[144]^ Bicontinuous structures in monoliths can be achieved by spinodal decomposition during the synthesis, thereby leading to a microporous monolith,^[145]^ or by coordination replication, which can create a range of mesostructured monoliths and materials through the transformation of a structured monolith consisting of, for example, a precursor metal oxide into a MOF while maintaining its mesostructural characteristics.[^71^, ^84^, ^146^]

## 8 Reticular Bulk Objects

Reticular materials are manufactured into bulk sizes to exploit their extensive properties arising from the sum of its parts, for example, absolute gas storage capacity, rather than the intensive properties. Real‐world applications require a considerable output of specific extensive properties, thus demanding the large‐scale use of reticular materials. Typical conditions for MOF and COF synthesis produce loose powders which exhibit undefined volumes and are challenging to process. Single crystal,[^99^, ^147^] monolith,^[148]^ pellet,^[149]^ and glass^[150]^ (Figure 4 g) are the physical forms favoured for application scenarios, with each form exhibiting a defined volume, a desired geometry, and a tuneable size. The specific physical form of the final reticular material is chosen based on the properties required for different applications. For example, transparency is found in a single crystal and glass; unidirectional mass transport can be enabled by films and pellets; complex architectures can be presented by a monolith. For structuring reticular materials on this macroscopic scale, various externally controlled tools have been developed, including casting, pressing, and printing. These techniques, in parallel with the chemical control exerted by reticular design, produce bulk reticular objects that display optimized proton and thermal conductivity, elasticity, and gas capture and storage capabilities for a wide range of applications (Figure 5).

Monoliths are key players in the industrial use of reticular materials (Figure 4 h). MOFs and COFs are capable of gas uptake with high gravimetric and volumetric capacities, exceeding the performance of other porous counterparts that have been made. Although the values of these parameters are typically obtained by measuring reticular materials in the form of single crystals or polycrystalline powders, real‐world applications require the same measurement undertaken with their monoliths.^[151]^ Whether it is methane storage in a car fuel tank or CO_2_ capture from industry‐scale pipelines, packing as much material in a given volume is one of the priorities. Therefore, interstitial space between particles and crystallites, which do not contribute to gas uptake, should be minimised. By structuring frameworks into a shaped monolith, typically with binders,^[152]^ empty space is significantly reduced and the density of the final material is increased. The shape of the monolith can be tuned to meet the specification of the container used.

An advanced level of structuring reticular materials is to shape the monolith into complex architectures. Catalytic converters epitomise the concept of using interconnected channels to decrease flow resistance and thus facilitate mass transport. Implementing architecturally optimized monoliths into a relevant application context promotes gas uptake and release kinetics.^[81b]^ Furthermore, reticular materials can be combined with other types of materials into one monolith. The hybrid construct will benefit the interplay between multiple material performances in a single process, for example, integrated water harvesting^[153]^ (Figure 5) and photothermal conversion.^[154]^ Finally, the architecture of the monolith determines its overall mechanical stability, and this aspect has to be taken into consideration to satisfy the need of the application environment.

The approaches used for the production of monoliths are largely based on externally controlled tools. The monoliths can be prepared by casting precursor mixtures into a desired shape in combination with wetting the solid precursors,^[155]^ evaporation,^[156]^ extrusion,^[157]^ a sol‐gel method^[158]^ or an organic terra cotta approach in COFs.^[159]^ Alternatively, pre‐synthesised powders of reticular structures are pressed into a monolith shape.^[160]^ The pressure used needs to be controlled within a range that does not lead to structural collapse of the framework structures.^[14]^ Pre‐existing monolith scaffolds^[161]^ and 3D printing[^78d^, ^81a^, ^81b^] techniques have been developed for constructing reticular monoliths of complex architectures. Additive manufacturing or 3D printing (Figures 3 and 4 f) of MOFs and COFs is challenging due to the necessity for rapid solidification of the product to be able to print it into monoliths. Several approaches to print 3D monoliths from reticular materials have been developed. A straightforward approach is the production of a gel from only the MOF precursors and solvent which can be directly printed.^[78d]^ By embedding pre‐formed materials in a shear thinning liquid that can be directly printed and do not flow freely anymore as soon as it has been deposited, the shear thinning liquid can be removed after the printing. This approach largely allows the retention of the porosity and enables high loading and low amounts of binder in the final monoliths. Often a type of gel composite is used here, such as one based on hydroxyethylcellulose.[^81a^, ^152g^, ^162^] Embedding the reticular material in a polymer matrix allows printing by the typical melt extrusion used in commercial 3D printers, but this approach does not allow high loading and a reduction in the porosity can be observed.^[163]^ In reactive printing, precursors are reacted in situ and solidification is used as the printing principle.^[164]^

In addition, soft substrates such as cloths, foams, and sponges, which exhibit both porosity and flexibility, have been employed for constructing reticular monoliths. Strategies to synthesise these materials include the introduction of MOF particles into a foam‐synthesis mixture,^[103]^ dip‐coating pre‐existing foams with pre‐synthesized nanoparticles,^[165]^ synthesizing MOF particles in the pores of foams,^[166]^ by nucleation of MOF or COF crystals on the fabric surface,[^12i^, ^167^] and by synthesising monoliths by coordination replication.^[168]^

Pelleting particles into a cylinder shape allows large lateral areas with small thicknesses to be achieved. This form has a shape that is similar to that of a membrane, which features an even smaller thickness. The reason for having a small dimension is to take advantage of the short mass transport pathway along this direction to make electron‐ or ion‐conductive materials. For example, crystals of an anionic framework have been mechanically pressed into a pellet to serve as a solid electrolyte for a lithium‐ion battery.^[169]^ As presented in this work and many other cases, when small crystals (powder) are available, pellets are the preferred form of final material, since the thickness can be tuned.

Glasses are amorphous structures that exhibit a disordered arrangement of their chemical building blocks. Similarly, reticular glasses show a lack of long‐range periodic order, while still preserving the short‐range order in the connection of their building units. The production of reticular glass occurs through the combined synthetic control from both self‐assembly and external tools (Figure 4). They can be synthesised directly by using modulating and high‐viscosity solvents;^[102]^ however, the majority of MOF glasses have been manufactured by the amorphization of their parent framework structures by applying high pressure^[170]^ or melting and quenching.^[171]^ Compared to their parent compounds, MOF glasses display transparency, improved mechanical stability, and an increased density,^[172]^ but can still remain microporous (Figure 5).^[173]^

## 9 Applications of Reticular Materials Benefiting from Structural Control

The application of reticular materials is highly dependent on their structuring across all length scales (Figure 5). With structural features deliberately controlled by self‐assembly and external tools, frameworks have been developed into materials that exhibit unique properties at every level.

The following section, therefore, describes the most important application fields for reticular nano‐, meso‐, and bulk objects.

## 10 Specific Applications of Nano‐Objects

Combining the versatile chemistry of MOFs and COFs with the properties of the nanoworld opens a door for a large variety of applications for nanoparticles.[^88^, ^174^] These properties include short diffusion paths, fast kinetics, and sorption properties, as well as size‐dependent optical, electrical, and magnetic properties.

As a result of their enhanced conversion rates and sorption kinetics, MOF nanoparticles find applications in fields such as catalysis^[175]^ and adsorption,^[89]^ as well as in electrical applications, for example, as composite materials in supercapacitors.^[176]^

Reducing the size of MOF particles down to the nanoscale to form Zr‐based MOF nanoparticles has led to a dramatic enhancement in the catalytic degradation of a nerve agent simulant.^[175]^ Significant rate enhancements were observed for the nanocrystals compared to the microcrystals of the same materials. These effects are due to the larger external surface area relative to the internal surface area and/or faster diffusion.^[175]^

Many reticular materials show flexibility or breathing during gas adsorption, where structural changes induced by the gas adsorption lead to dramatic changes in the ability to take up gases. These phenomena are typically cooperative phenomena that are significantly affected by particle size. Therefore, the particle size can be used to tune gas adsorption and cooperative adsorption features in particles of reticular materials. It was observed that the gate‐opening pressure shifts with the crystallite size,^[177]^ thus indicating that particle size can be used to tune the gate‐opening for specific pressure ranges and applications. The crystallite size also affects negative gas adsorption/pressure amplification.^[132b]^ In these cases, a complete suppression of the cooperative adsorption phenomena is also observed for sufficiently small crystallites.^[177]^

Reducing the diffusion length by increasing the external surface area through the use of nanosized particles is also beneficial for electrode materials for batteries and supercapacitors. In these cases, the faster diffusion rates lead to improved rate capabilities and higher deliverable capabilities, as smaller particles increase the contact with the electrolyte and the device benefits from shortened electron and ion transport pathways.[^176^, ^178^] The shortened diffusion lengths can also increase the utilization of electroactive components, such as sulfur in lithium sulfur batteries, where a faster and more efficient conversion is possible with smaller sizes.^[179]^ Although a decreased particle size might lead to a further increase in diffusion and utilization, a compromise with other device characteristics is necessary, such as the stability or cycling performance of the device.^[176]^

The main focus in the application of MOF and COF nanoparticles lies in the field of biomedicine (Figure 5).^[180]^ Nanosized particles are favourable in biomedical applications as they exhibit improved endocytosis and are well‐dispersed in body fluids. Reticular nanoparticles are investigated in this field because of their high internal surface area and functionalizability. Both these features can be tuned through control of the inner pore wall by reticular chemistry. This allows the precise tuning of host–guest interactions and ultra‐high porosity, both of which are central for the high loading and release of active molecules.[^28a^, ^36^, ^181^] Reticular chemistry allows not only to use reticular nanoparticles as transport vehicles, but enables their scaffolds to be turned into active components for cancer treatment,^[182]^ by providing linkers as active components^[180c]^ or with metal species that affect cell growth.^[183]^ The MOF nanoparticles can also exhibit optical and catalytic properties at the targeted site in vivo to help treat cancer, by catalyzing the formation of reactive oxygen species for photodynamic treatment.^[184]^

Straightforward functionalization of the external surface of reticular nanoparticles facilitates cell uptake and can be used to introduces targeted functional groups for cell uptake, or even be used to deliver drug molecules.^[31]^ External functionalization of reticular nanoparticles also affects the stability in vivo and enables the breakdown and application of their function only upon uptake by the cell.^[183]^

In addition to therapeutics, reticular nanoparticles can be used for different biomedical imaging techniques (e.g. positron emission tomography, magnetic resonance imaging)[^27b^, ^185^] or in combination with drug delivery.^[186]^

Endowing a reticular material with biocompatible building blocks to minimize toxicity, makes MOF nanoparticles ideal candidates to utilize in vivo.^[97b]^ The size of MOF nanoparticles enable easy uptake by the cell, which can be enhanced further by coating strategies, such as polymer or lipid coatings.[^27b^, ^62b^, ^187^]

Luminescent functionalization allows the MOFs to be used as chemical sensors,^[188]^ with easy monitoring of their pathways and metabolism within living cells.^[189]^ Furthermore, the tailorable nature of MOFs allows the use of biocompatible building blocks and their combination with the hybrid composition of the MOF scaffold result in high biodegradability and biocompatibility, as the toxicity of MOF nanoparticles can be influenced by the choice of the metal and organic component, independently.^[190]^ Importantly, the toxicity of nanoparticles also highly depends on size, shape, surface area, surface charge, and dose and cannot be limited to composition only (Figure 5).[^92^, ^191^]

In comparison to MOFs, COFs benefit from their metal‐free nature and avoid incompatibility with cells because of the presence of heavy or toxic metals.^[192]^ The cytotoxicity and water instability of MOFs limit their cellular and biological applications, when a fast breakdown of the framework is not desired.^[193]^ MOFs can, therefore, be used to facilitate the release of active species by degradation of the MOF itself. These features can be overcome by using COFs for drug delivery and applications where biocompatibility is required.[^36^, ^181g^, ^192^]

## 11 Specific Applications of Meso‐Objects

Applications of mesostructures rely on the strong hierarchy in their respective systems. 0D MOF and COF mesostructures offer both compartmentalization and a large external surface area for functionalization, which is why they are mainly examined for catalysis,[^112a^, ^112b^] drug delivery,^[194]^ and in ion conduction^[118]^ (Figure 5). Similarly, 3D reticular mesostructures can be used as precursor materials for the generation of sophisticated catalysts. Additionally, assemblies of 3D mesostructures have been used for filtering applications^[195]^ and in sensors based on a change in the optical properties of the materials upon guest uptake.^[196]^ The majority of the applications of COF and MOF mesostructures, however, are film‐based. Here, 1D spatial confinement results in reduced transport distances for electron and ion conduction. Additionally, mesomaterials provide an increased interface between a surface and its surrounding medium, which is beneficial for catalytic applications. The growth of MOF and COF films on supports is a straightforward method to augment a carrier material with advantages such as porosity, crystallinity, ion conductivity, and new optical properties. These properties make the thin‐film framework materials interesting for sensing applications.^[197]^ The porous nature allows the inclusion of guest molecules within the frameworks, which then cause, for example, changed absorption spectra and altered refractive indices that can be read out, thereby leading to potential uses in the detection of small molecules.^[198]^

Porosity, a high interface with the surrounding medium, and a high concentration of reactive sites are reasons for the use of framework films in catalysis.^[199]^ Similarly, a framework surface coating can enhance the reactivity of electrodes by facilitating charge‐carrier separation and providing enhanced light absorption for application in water splitting.^[200]^ The surface can also template a crystalline co‐orientation of the resulting thin film and result in enhanced catalytic performance for the electrocatalytic reduction of CO_2_, where facile charge transport is enhanced by alignment.[^24a^, ^124a^]

Framework thin films have also been examined for ion conduction such as with lithium and protons: The porous structure of MOFs can either be functionalized with proton‐conducting dangling groups on the surface, such as sulfonate moieties, or be loaded with proton‐conducting guest molecules such as imidazole and histamine.^[201]^ Furthermore, thin films composed of COF nanosheets have been used for lithium‐ion conduction and as separators in batteries.[^38c^, ^202^]

The combination of a porous structure and large interface area of 2D reticular mesostructures is strongly related to their applications in water purification^[203]^ and gas separation.^[204]^ Films of MOF nanosheets can selectively filter mixtures of gas molecules containing molecules such as H_2_ or CO_2_. Selection criteria include the size of the gas molecules or their chemical nature, as they have to interact with the pores of the material. This advantage can be further emphasized through post‐modification of the pore size or the introduction of functional groups such as amino groups into pores to further improve the selectivity. Gas separation based on mixed‐matrix membranes (MMMs) composed of either COF particles or nanosheets has been used in COFs, where a binder is used to improve the processability of the COF.[^152d^, ^152e^, ^152f^, ^205^]

In the past, MOFs have been understood as not being suitable as electron conductors because of their metal centres generally preventing electron resonance delocalization and the lack of redox‐active ligands. This is slowly changing with the development of new electron‐rich linker molecules, the inclusion of guest molecules, and the development of MOFs with “through‐space” mechanisms, such as π–π stacking, which have opened up the use of MOF films for such applications.^[206]^ Frameworks with improved charge mobility have also been realized by the synthesis of mixed‐valence frameworks^[207]^ and the introduction of charge‐transport pathways through heteroatoms such as sulfur.^[208]^ In contrast, COFs have often been used as semiconducting materials, as most demonstrated COFs are two dimensional, with π–π stacking in the third dimension that facilitate charge transport. In many 3D COFs, extensive π–π contacts are even present due to interpenetration.^[209]^ The π–π interaction facilitated by the stacking allows for facile charge conduction.^[210]^ The in‐plane conductivity is often hampered in COFs by the intrinsic polarization of the COF linkages, such as in boronate COFs and imine‐based COFs. However, recent developments in the development of new COF linkages has improved the in‐plane conductivity through the use of linkages such cyanovinyl or phenazine linkages.^[211]^

The porous nature of reticular materials makes them ideal materials for sensing applications, as their porosity enables access of analytes to the inside of the material and thus, in contrast to dense materials, not only is the outside surface of the materials active but also the inside surface. Sensing applications often requires a specific mesostructure that is highly dependent on the fundamental sensing principle. Thin films are needed for sensing changes in electrical and ionic conductivity,^[212]^ mass changes in a quartz microbalance,^[105a]^ or changes in the optical properties of Fabry–Perót devices.^[213]^ The porous nature of MOFs and COFs in combination with conductivity present an ideal case for sensing applications, since the conductivity allows for facile readout of the signal (Figure 5). Electrical conductivity is used to detect a change in the resistivity of a sample in response to a stimulus, such as gas molecules that can be detected with MOFs or COFs.[^211b^, ^212^, ^214^] Electrical conductivity and structuring as thin films is also necessary for applications in electrocatalysis,[^24a^, ^215^] for supercapacitors,[^210c^, ^216^] photovoltaic devices,^[217]^ and for photoconductivity.[^33a^, ^218^]

More complex architectures are constructed for sensing based on responsive photonic crystals (Figure 5). In these cases, one‐, two‐, or three‐dimensional periodic structures with a high degree of regularity need to be generated to achieve good optical properties. Since the sensing principle of photonic crystals is based on general properties, such as the refractive index of the analyte and the uptake in the porous structure, photonic crystals are generally applicable to sensing applications and, therefore, highly sought after. One‐dimensional photonic crystals can be constructed from MOFs by constructing optical lattices of repeated layers of thickness‐controlled thin films of the MOF and either a second inert layer or a second MOF layer with different adsorption properties, which can be constructed by spin‐coating nanoparticle suspensions, or by growing MOF thin films in alternation with titanium dioxide layers.^[122]^ 3D photonic crystals are also called opals and possess periodicity in three dimensions; these can be constructed from MOFs by coordination replication of a metal precursor lattice that has been templated with polymer beads to have 3D periodicity, where the metal precursor lattice gets transformed into a MOF by reaction with a linker solution^[84]^ or by filling the voids in a pre‐assembled opal of polymer particles.^[198]^ A different approach is the synthesis of highly size‐ and habit‐controlled MOF nano‐ or microparticles, which are then assembled into 3D crystallite superlattices, which possess sufficient domain size and low polydispersity as well as sufficient optical properties to allow the direct optical sensing of guest molecules in the pores (Figure 4 c).^[100]^

## 12 Specific Applications of Bulk Objects

Reticular materials that are used in bulk‐scale applications rely mainly on properties that originate from their framework structure. The extension of the dimensions of the frameworks to materials in the bulk regime amplifies the physical behaviour of a unit cell in the collective performance of the whole material. These applications require a specific macroscopic shape, such as columns and reaction beds, into which framework powder can be packed.

MOF and COF bulk materials offer diverse applications in heterogeneous catalysis. For one thing, their porous structure allows the loading of catalytic active species such as metal nanoparticles[^24b^, ^219^] or single‐site metal complexes^[220]^ into their pores, thus making MOFs and COFs excellent carrier materials. The heterogeneous build‐up of the scaffold structure can provide catalytic active centres through both the metal centres^[221]^ as well as the linker molecules[^55c^, ^222^] or the COF linkages.^[223]^ The precisely defined pores of a MOF and COF structure deliver their catalytic potential by providing the material with a structural selectivity towards both starting materials and products, filtering molecules by size[^223^, ^224^] as well as chirality.^[225]^

New gas storage technologies are readily sought after as they are especially relevant in fuel technology. Materials that are used in this field need to exhibit a combination of high storage capacities, high cycling stability, low adsorption/desorption enthalpies, a high thermal conductivity, and high safety (Figure 5). Fuel tanks based on COFs and MOFs can meet many of these criteria: the adsorption of gas molecules such as hydrogen^[226]^ and methane[^226b^, ^227^] into the porous structures allows high volumetric storage capacities at reduced pressures, which results in easier refuelling and increased safety because of the lower pressurization of the fuel container.

Their porous nature has also opened up the field of heat transfer to MOFs. Heat in MOFs is transported primarily along the chemical bonds of their porous structure. The low atomic density of their crystal structure results in a low thermal conductivity (around 0.1 W m^−1^ K).^[228]^ Here, structures act as heat pumps that distribute energy through the adsorption and desorption enthalpies of small molecules.^[229]^ Research in this field is most advanced in the case of water harvesting, where MOFs are already surpassing benchmark materials.^[230]^ Similarly, these sorption properties have resulted in MOFs being used in air dehumidification.^[231]^ Species with a sufficiently small desorption enthalpy can even be used to extract water from air through solar powering.^[232]^

## 13 Outlook

The synthetic effort made by reticular chemists has been largely focused on periodic, crystalline structures, which has led to the discovery of frameworks of new structure types and the use of such structures for specific purposes. We encourage a shift in the attention from crystal structures towards their morphological manifestation at the final stage. This requires raising the awareness of how much the physical forms that the reticular materials are structured into can affect the properties relevant to application scenarios.^[233]^ This aspect has been taken into consideration when volumetric gas adsorption capacity is calculated using the geometrical volume of the pellet, instead of using the one calculated from the crystallographic density.^[158]^ We hope that such a consideration can permeate into many other application fields, so that the parameters of reticular materials are measured and compared in a way that is directly expressed in a practical setting. In many other applications, anisotropic properties displayed by framework materials are valued, for example, electron and ion conductivity, which are much more dependent on the orientation, arrangement, and distribution of reticular crystals and the chemistry of their interfaces. It is widely observed that a single crystal of high conductivity does not simply guarantee the conductivity of the membrane in which it is incorporated. As such, there remains much to be explored for structuring reticular materials beyond their unit cells to excel for specific tasks.

Manufacturing reticular materials in the nano‐, meso‐, and bulk regimes can be facilitated by self‐assembly or external control; at each scale, the importance of these two approaches are weighted differently and a specialised set of methods have become available. We believe the research on using external tools to structure reticular materials will continue to expand, especially those tools that work in parallel with self‐assembly for achieving advanced structure control. However, such a cooperation is applicable only in a relatively narrow range of size, typically spanning not more than one to two orders of magnitude (Table 1) and mostly converging at the mesoscopic regime. In the history of discovery, every time that chemical and physical control have been combined together through the advent of a technique (e.g. layer‐by‐layer growth),^[85b]^ a new field of study and a previously unexplored landscape of chemical structures has emerged.^[234]^ We expect that the interplay between chemical and physical structuring will yield new results for reticular materials, considering the high level of chemical control expressed by the linking of molecular building units.

We envision that the development of external tools will enable that even larger length scales could be addressed when shaping reticular materials. Framework structures that can be made into monoliths in sizes of centimetres or even decimetres are appealing, as they could be directly used as a fuel tank in a car, a small‐scale catalytic converter, a water‐harvesting chamber, structural materials for heat insulation and acoustic absorption, paint for modulating humidity, or window glasses for formaldehyde photo‐degradation. To achieve manufacturing capability at this level, external tools other than mechanical shaping and templating (the two physical methods mostly used so far) are needed for structuring reticular materials. Potential candidate techniques are two‐photon polymerization (for photo‐induced linkage chemistry), ion‐beam deposition (with ionic building units), advanced 3D printing, and etching processes compatible with linkage chemistry.

## Conflict of interest

The authors declare no conflict of interest.

## Biographical Information

*Frederik Haase obtained his PhD with Prof. Bettina V. Lotsch from the University of Munich (LMU, Germany), while working at the Max‐Planck Institute for Solid State Research (Stuttgart, Germany). He then worked as a postdoctoral fellow with Prof. Shuhei Furukawa at Kyoto University (Japan), funded by the Japan Society for the Promotion of Science. His research is focused on designing and understanding assembly pathways in covalent organic frameworks and metal‐organic frameworks*.

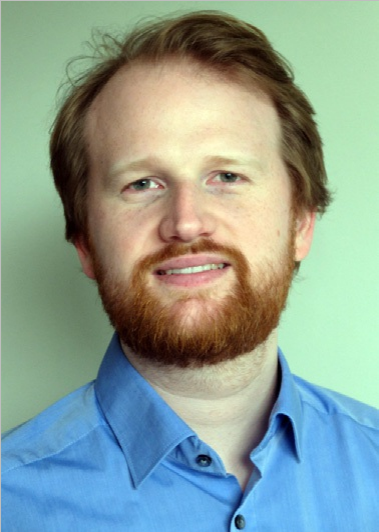

## Biographical Information

*Zhe Ji completed his bachelor's degree at Tsinghua University with Prof. Gaoquan Shi, and then moved to the group of Prof. Omar M. Yaghi at UC Berkeley, where he completed his PhD on the design and synthesis of reticular materials. He is currently a postdoctoral researcher at Stanford University in the group of Prof. Steven G. Boxer. His research interests lie in bringing a high level of complexity and functionality to framework structures by connecting metal‐sulfur clusters, sequencing metals in multivariate systems, and building framework‐bacteria interfaces*.

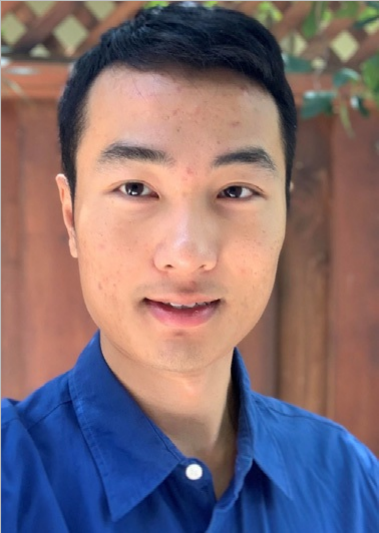

## Biographical Information

*Stefan Wuttke created the research group “wuttkegroup for science”, initially hosted at the Institute of Physical Chemistry at the University of Munich (LMU, Germany). Currently, he is an Ikerbasque Professor at the Basque Center for Materials, Applications and Nanostructures (BCMaterials, Spain). His research is focused on developing methods to write and read chemical information onto and from the backbone of hybrid framework materials*.

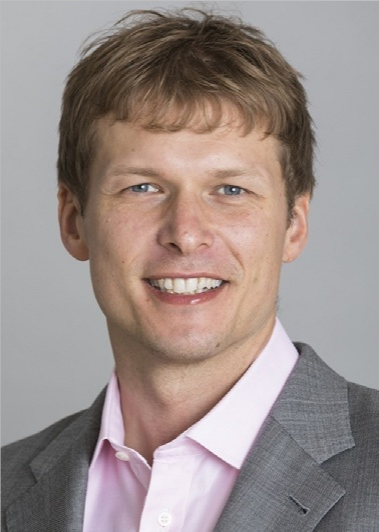

## Biographical Information

*Ralph Freund is currently a PhD student at the University of Augsburg (Germany) under the supervision of Prof. Dirk Volkmer. He obtained his MSc in chemistry from the University of Munich (LMU, Germany) with Stefan Wuttke in 2018, investigating the chemistry of metal oxide to metal–organic framework conversion reactions. His current research focuses on electric‐field‐responsive porous coordination frameworks constructed from pyrazolate linkers*.